# Evidence That Artificial Light at Night Induces Structure-Specific Changes in Brain Plasticity in a Diurnal Bird

**DOI:** 10.3390/biom11081069

**Published:** 2021-07-21

**Authors:** Stan Moaraf, Rachel Heiblum, Monika Okuliarová, Abraham Hefetz, Inon Scharf, Michal Zeman, Anat Barnea

**Affiliations:** 1 Faculty of Life Sciences, School of Zoology, Tel-Aviv University, Tel-Aviv 6997801, Israel; hefetz@tauex.tau.ac.il (A.H.); scharfi@tauex.tau.ac.il (I.S.); 2 Department of Natural and Life Sciences, The Open University of Israel, Ra’anana 43710, Israel; rachel.heiblum@gmail.com (R.H.); anatba@openu.ac.il (A.B.); 3 Department of Animal Physiology and Ethology, Faculty of Natural Sciences, Comenius University, 84215 Bratislava, Slovakia; monika.okuliarova@uniba.sk (M.O.); michal.zeman@uniba.sk (M.Z.)

**Keywords:** artificial light at night (ALAN), brain plasticity, cell proliferation, new neuronal recruitment, melatonin, sex-differences, birds, zebra finches (*Taeniopygia guttata*)

## Abstract

We recently reported that artificial light at night (ALAN), at ecologically relevant intensities (1.5, 5 lux), increases cell proliferation in the ventricular zone and recruitment of new neurons in several forebrain regions of female zebra finches (*Taeniopygia guttata*), along with a decrease of total neuronal densities in some of these regions (indicating possible neuronal death). In the present study, we exposed male zebra finches to the same ALAN intensities, treated them with 5′-bromo-2′-deoxyuridine, quantified cell proliferation and neuronal recruitment in several forebrain regions, and compared them to controls that were kept under dark nights. ALAN increased cell proliferation in the ventricular zone, similar to our previous findings in females. We also found, for the first time, that ALAN increased new neuronal recruitment in HVC and Area X, which are part of the song system in the brain and are male-specific. In other brain regions, such as the medial striatum, nidopallium caudale, and hippocampus, we recorded an increased neuronal recruitment only in the medial striatum (unlike our previous findings in females), and relative to the controls this increase was less prominent than in females. Moreover, the effect of ALAN duration on total neuronal densities in the studied regions varied between the sexes, supporting the suggestion that males are more resilient to ALAN than females. Suppression of nocturnal melatonin levels after ALAN exhibited a light intensity-dependent decrease in males in contrast to females, another indication that males might be less affected by ALAN. Taken together, our study emphasizes the importance of studying both sexes when considering ALAN effects on brain plasticity.

## 1. Introduction

Artificial light at night (ALAN) disrupts the daily light–dark cycle and exposes animals and humans to higher levels of nocturnal light. It has vast biological impacts on many species, and is also associated with several health problems in humans [1,2]. For example, ALAN has been shown to induce earlier reproductive maturity [3,4], compromise immune homeostasis [5], and decrease melatonin (MEL) production [6,7]. Nevertheless, little is known regarding the effects of ALAN on brain plasticity processes such as cell proliferation (CP) and new neuronal recruitment (NR), and most of the existing studies have been conducted on nocturnal mammals, e.g., [8,9,10,11]. Therefore, in a previous study we investigated this question in diurnal female zebra finches (*Taeniopygia guttata*), by exposing them to three weeks of ecologically relevant ALAN intensities (0.5, 1.5, and 5 lux), and found that ALAN increased CP in the ventricular zone (VZ) in the brain as compared with controls that were kept under fully dark nights [6]. In birds, many of the new cells that are born in the VZ migrate into the telencephalon, differentiate into neurons, and settle in various brain regions, where they replace older ones [12,13] and are recruited into functional circuits [14,15]. Therefore, in that study we also recorded neuronal densities in several forebrain regions, and found that despite the increased proliferation, total neuronal densities decreased in some regions, suggesting net neuronal death. In a later study [7], in which we exposed female zebra finches to a longer ALAN exposure (six weeks), we recorded NR in these brain regions and found that ALAN increased recruitment compared to controls, possibly as a compensatory response to ALAN-induced neuronal death, and/or due to increased nocturnal locomotor activity caused by sleep disruption. In addition, the decrease in total neuronal densities, which we observed in some brain regions under short-term ALAN exposure [6], was not found under the longer exposure [7], indicating a temporal effect of ALAN.

Our previous studies were conducted on females. Therefore, the aim of the present study was to test whether the observed effects of ALAN on neuronal plasticity occur also in males. Moreover, a major advantage of studying males is their unique song system, which does not exist in females [16] and is known to contain MEL receptors, e.g., [17]. This enabled us to specifically test the effects of ALAN on the song system. Two nuclei from this system were included in our study: (1) HVC, which is involved in the control of vocal behavior, and incorporates new neurons into functional circuits [14,18], and (2) Area X, which exhibits large-scale neuronal addition throughout adulthood, is critical for the acquisition of song in juveniles [16,19], and plays a role in song maintenance in adults (reviewed in [20]). It is well established that female mate choice in many songbird species is influenced by the male singing behavior, which can serve as an indicator of his quality [21]. Therefore, an effect of ALAN on the neuronal composition of the song nuclei could result in the production of an impaired or mistimed song, which in turn might have male-specific fitness consequences by decreasing the males’ ability to attract or retain a mate and defend a territory, and hence might have ecological implications. In addition, our present study, which focused on males, enabled us to add a comparative dimension, specifically in regard to looking for possible sex-differences in the response of the brain to ALAN. Testing this question is important because at least some aspects of neuronal plasticity, such as CP, might differ between sexes, e.g., [22,23].

To achieve these aims, male zebra finches were exposed to ALAN intensities of 1.5- and 5 lux for three and six weeks and compared to controls that were kept under dark nights. We recorded CP in the VZ, as well as NR and total neuronal densities in two song nuclei HVC and Area X. In addition, we recorded the same neuronal parameters in three other forebrain regions, which were also tested in our previous studies with females: the nidopallium caudale (NC), which contains auditory relays [24] and is involved in vocal communication and the integration of auditory information [25,26]; the medial striatum (MSt), which is a part of the avian somatomotor basal ganglia [27] and is linked to visual perception and associative learning [28,29,30]; and the hippocampus (HC), which processes spatial information [31,32] and plays a role in stress response [33].

## 2. Materials and Methods

### 2.1. Experimental Design

**Figure 1:**
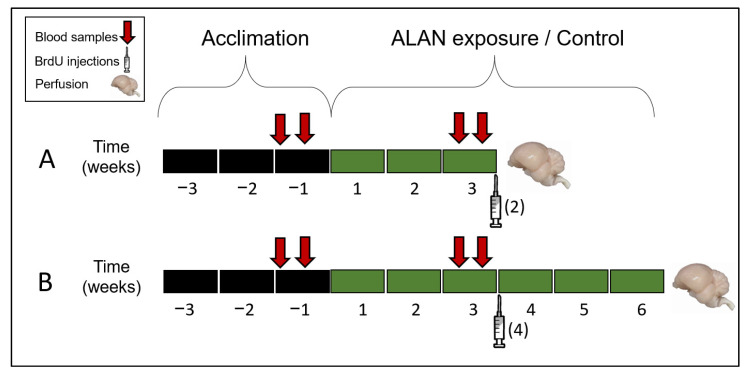
A schematic description of the experimental design. (**A**) Timeline of the cell proliferation experiment. (**B**) Timeline for the neuronal recruitment experiment. Red arrows indicate blood sampling; syringes indicate BrdU treatment times; and the numbers in brackets indicate the number of injections. See text for details.

**Figure 2:**
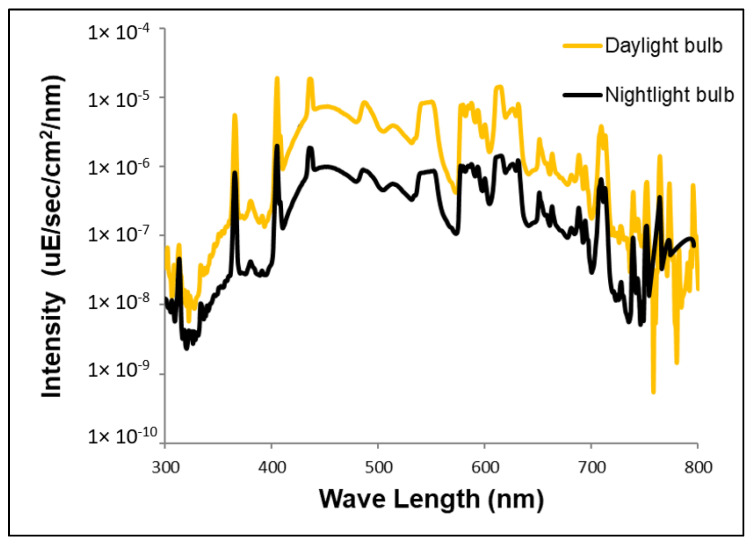
The spectrum and intensity of each light source that was used in the experiment for day and night-time illumination.

Our study consisted of two experiments, a **cell proliferation (CP) experiment**, which included three groups with six male birds per group (Figure 1A), and a **neuronal recruitment experiment (NR)**, which included two groups with eight male birds per group (Figure 1B). All of the following experimental conditions and procedures were identical to those in our previous studies with females [6,7]. Zebra finches were hatched and reared in our outdoors breeding colonies, at the I. Meier Segals Garden for Zoological Research at Tel-Aviv University, Israel. Sexually mature males (ranging between three to five months of age, out of a lifespan of four to five years [34] for all groups) were transferred, one group at a time, from their native colonies to a room, in which day length and temperature were set to 14:10 L:D and 27 °C. Zebra finches are very social birds, and in order to avoid isolation stress, birds of each group were kept together in one cage (67 × 33 × 33 cm). The cage was placed in the same location within one room, which was used for all the groups for the entire duration of the experiment. In order to ensure constant and identical conditions within and between groups, temperature, moisture and light intensities were monitored every 10 days at the same locations around and in the cage. After the experimental birds were transferred from their native colonies to indoor conditions, they were all given three weeks of acclimation, a period that ensures thermogenic acclimation [35] and is accepted for physiological acclimation in birds, e.g., [36]. During the acclimation periods, the birds were exposed to a full spectrum daylight fluorescent light source (1200 lux; CFL, Hyundai, Macedonia) during the day, and were kept in complete darkness during the night. Following the acclimation period, all birds were still exposed to the same light source during the day, as described above. During the night, for the ALAN groups, we used the same type of light source that was partially shaded, so that birds in the **CP experiment** were exposed either to **1.5- or 5lux** ALAN intensities for three weeks (Figure 1A) and birds in the **NR experiment** were exposed to **5lux** ALAN for six weeks (Figure 1B). The cage contained four perches on which the birds could stand and sleep. ALAN intensities were measured at all these perches in order to ensure that the birds were exposed to constant ALAN intensities, regardless of their location in the cage. **Control** groups were exposed to dark nights for the duration of the respective experiment. Spectra of all the light sources were measured by a spectrometer (Jaz spectrometer, Ocean Optics, Largo, FL, USA) and intensities by a digital light meter (TES-1337, TES, Taipei, Taiwan), to ensure full and uniform spectra and intensities (Figure 2). For the entire duration of the experiment, food (millet seeds) and water were provided ad libitum three times per week. As an indirect indication of the birds’ health, each bird was weighed (Precisa, BJ 120 °C ± 0.01 g) seven times (three times before and four times during ALAN exposure), every week, four to six hours after lights were turned on. We also measured MEL levels in the birds’ plasma, and after three weeks of the ALAN period treated them with 5′-bromo-2′-deoxyuridine (BrdU), which is a cell birth-date marker (Figure 1 and see below).

### 2.2. Melatonin Levels in Plasma

Four blood samples were taken from each bird (Figure 1): at midday on day one of week three of acclimation, at midday on day one of week three of the ALAN period; and two at midnight, three days after each of the midday samples, to allow enough time for the birds to recover between bleedings. Blood collection at midnight was performed as described in [7]. Plasma MEL concentrations were measured by direct radioimmunoassay [37], which has been validated for zebra finches [38]. For detailed description see [6]. We used specific MEL antiserum (Stockgrand Ltd., G/S/704-8483, University of Surrey, Guildford, UK) and [O-methyl-3H]-labelled melatonin (specific activity: 3.07 TBq/mmol, Perkin Elmer, Waltham, MA, USA). Sample radioactivity was measured in the scintillating β-counter for liquid samples (Packard Tri-Carb 2900 TR, Packard Instruments, Perkin Elmer, Waltham, MA, USA). All samples in each experiment were measured in a single assay with intra-assay variation coefficients less than 10.0% and the assay sensitivity 0.5 pg/tube.

### 2.3. BrdU Administration and Immunohistochemistry

To study the effect of exposure to ALAN on CP in the brain, all birds from the CP experiment were treated with BrdU (SigmaUltra) at the end of the three weeks of ALAN exposure (Figure 1). Two intramuscular injections of 130 µL of BrdU (diluted 10 mg/mL in sterile water; for details see [39]) were administered within 24 h, one at 15:00 on the day before the perfusion, and the other at 07:00 on the perfusion day. Birds were killed two hours after the second injection. This protocol enabled labelling of new cells in the VZ before they start their migration [40]. Histology and immunohistochemistry were performed as described in ref. [39].

**Figure 3:**
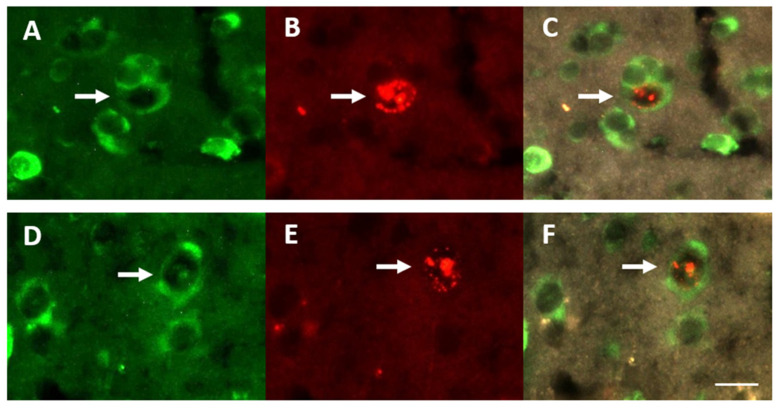
New neurons shown in three microphotographs of the same field, in the Medial Striatum (MSt; **upper row**), and the nidopallium caudale (NC; **lower row**) in brains of zebra finches (*Taeniopygia guttata*). HU-labelled neurons are shown under a FITC filter (**A**,**D**), and BrdU-labelled cells are shown under a rhodamine filter (**B**,**E**). The combined images were obtained using a FITC-rhodamine filter to show co-localization of the two markers (**C**,**F**). Scale: 10 µm.

To study the effect of exposure to ALAN on NR in the brain, all birds from the NR experiment were treated with four injections of BrdU, administrated within a 48 h period, two in the mornings and two in the afternoons, at three weeks of ALAN exposure. Birds were killed three weeks after the last injection. This protocol enabled labelling of new neurons within several brain regions (see below). Histology and immunohistochemistry for the NR experiment were performed as described in [7,41]. This staining protocol yielded neurons that were stained fluorescent green (with anti-Huc/HuD), and nuclei of new neurons that were stained fluorescent red (with anti-BrdU) (Figure 3). Therefore, cells with colocalization of green cytoplasm and red fluorescent nuclei were identified as new neurons. Accordingly, we could record the location of BrdU^+^ neurons and count them in each section, as explained below.

### 2.4. Mapping and Quantification

**Figure 4:**
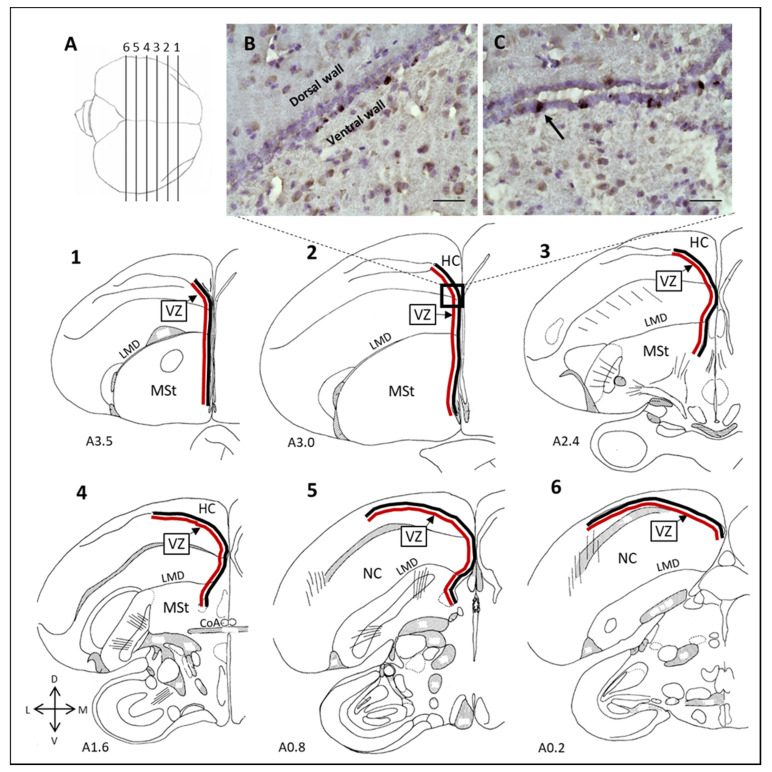
Mapping of cell proliferation in the ventricular zone (VZ). (**A**) Top view of a zebra finch brain (caudal is to the left, rostral is to the right). The numbered lines indicate the locations of the mapped sections, and frontal schematic illustrations of these sections are shown in 1–6 (taken from the atlas of the canary brain [42]). Red lines indicate the ventral walls of the VZ in each mapped section and the black lines indicate the dorsal walls. (**B**,**C**) Pictures showing two different small parts of the two walls of the VZ, to demonstrate new-born cells labelled with BrdU and stained dark brown (only one of the labelled cells is marked by an arrow). CoA, anterior commissure; HC, hippocampus; LMD, lamina medullaris dorsallis; MSt, medial striatum; NC, nidopallium caudale. Scale bar = 20 μm. (Adapted with permission from Ref. [6]. Copyright 2021, JOHN WILEY & SONS, INC.).

In the CP experiment, we used a computerized brain-mapping system (Neurolucida; Stereo Investigator; Micro-BrightField Ltd; Williston, VT, USA) to determine the number of labelled cells/mm in each of the VZ walls, and followed the protocol as described in [39]. We mapped six sections along the rostral-caudal axis of the VZ, at 720 μm intervals (Figure 4). The first section that contained the anterior commissure (CoA; corresponding to A1.6 in the atlas of the canary brain) [42] was determined as the fourth section. In this way, we ensured that the VZ location of each sampled section that was mapped along the rostral-caudal axis will correspond in all brains. In each section, the entire lengths of the ventral and the dorsal walls of the VZ were measured (indicated by the red line and the black line respectively in the 1–6 sections in Figure 4), and the number of BrdU-labelled cells that were found along each wall was divided by the length of that wall. This yielded the number of labelled cells/mm in each of the VZ walls.

**Figure 5:**
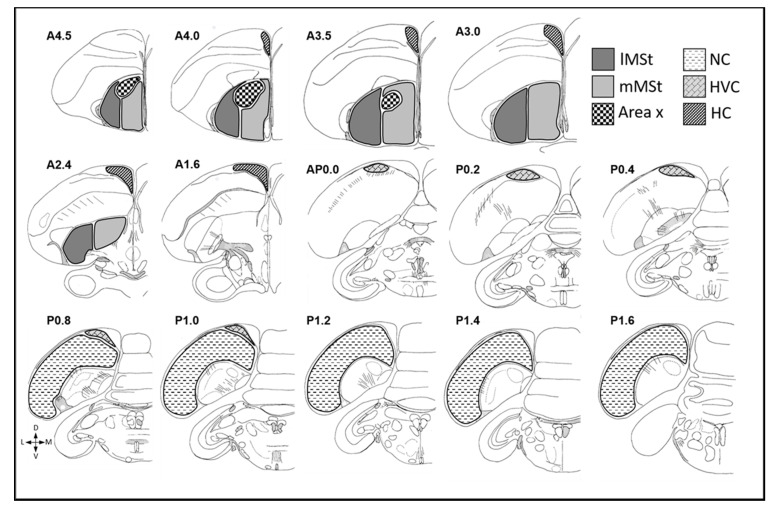
Mapping of new neuronal recruitment in several brain regions are shown. Illustrations of the mapped sections of medial striatum (MSt), nidopallium caudale (NC), hippocampus (HC), HVC and Area X are adapted with permission from Ref. [41]. Copyright 2021, Elsevier. Five sections of the lateral MSt (lMSt) and medial MSt (mMSt) (A4.5—A2.4), five sections of the NC (P0.8—P1.6), five sections of the HC (A4.0—A1.6), five sections of the HVC (AP0.0—P1.0) and three sections of Area X (A4.5—A3.5) are highlighted.

In the NR experiment we counted BrdU^+^ neurons in several brain regions that are known to recruit new neurons and represent various functions: the MSt, which was divided into its two sub regions, the lateral MSt (lMSt) and the medial MSt (mMSt); the NC; the HC; the HVC; and Area X (Figure 5). In each brain region we sampled five sections. In the MSt, the most rostral section corresponded to A4.0 in the canary atlas [42], and the fifth and most caudal section corresponded to A2.2. The MSt sub regions, the lMSt and the mMSt, were separated by drawing a straight dorsal-ventral line, at equal distances to the most lateral and medial boundaries of MSt. In the NC, the middle section corresponded to P1.2 in the canary atlas [42], and two more sections were obtained, rostrally and caudally, respectively, at distances of 360- and 600 µm from the middle one. In the HC, the most rostral section corresponded to A4.0 in the canary atlas [42], and the fifth and most caudal section corresponded to A0.2. The three other in-between sections were evenly distributed so that, on average, the distance between each mapped section and the next was 480 µm. In the HVC, the most rostral section corresponded to AP0.0 and the fifth and most caudal section corresponded to P0.8 [42]. In Area X, the most rostral section corresponded to A4.5 and third most caudal section corresponded to A3.5 [42]. In the MSt, HC, HVC and Area X, the middle sections were selected for measurements of neuronal density (see below).

We used the above-described brain-mapping system to draw the boundaries of each of the sampled brain regions and subregions in each of the five sampled relevant sections, to mark the position of BrdU^+^ neurons, and to quantify other neuronal parameters (see below). Mapping was done by using a 60× objective. Because a previous study from our lab [43] did not reveal any hemispheric differences in the density of labelled neurons in any region in males, we mapped sections only from the right hemisphere. In the lMSt and the mMSt, and in the HC, HVC and Area X, which are relatively small regions, we scanned the whole area in each mapped section by using the meander scan probe in our mapping system. However, since the NC is a large brain region, we scanned about 35% of the whole NC area in each mapped section as previously done in [44].

We also estimated the total neuronal densities (labelled and unlabeled neurons) in each of the sampled brain regions and subregions, following our previous protocol (see [45]). This was done in additional set of slides, with sections from the same locations as the slides in the above sets (6 µm apart), stained with cresyl violet, which reliably identifies neurons, e.g., [46]. This cresyl staining procedure was identical to the staining preformed in the CP experiment (see above), only without the addition of any antibodies. Finally, in each brain, and for each region, we also measured nuclear diameters of 10–15 BrdU^+^ neurons, as described in [47], and calculated the mean ± SE. This variable was used in the calculation of the Abercrombie stereological correction equation [48] to accurately estimate the number of BrdU^+^ neurons per mm^3^.

### 2.5. Statistical Analysis

All analyses were performed using JMP^®^, Version 14 software, (SAS Institute Inc., Cary, NC, USA, www.sas.com). CP and NR data were square-root transformed and those of MEL levels were log transformed to achieve normal distribution (Shapiro–Wilk test). Data were analysed using a repeated measures full-factorial mixed model, with ALAN as the between-subjects fixed factor and either section number, VZ wall, or brain region as the within-subject fixed variable. MEL levels for all groups were analysed by repeated measures mixed model analysis with ALAN as the between-subject factor and LD phase (day-before, night-before, day-during, and night-during) as the within-subject fixed variable. Bird identification was used as the random variable and Toeplitz covariance structure was used in the mixed model analyses. Individual one-way analyses followed whenever a significant interaction was found. Tukey’s post-hoc test was conducted to determine specific differences. Comparison of data between sexes were analysed using a mixed model with sex as a the between-subjects fixed factor.

## 3. Results

### 3.1. ALAN Increases Cell Proliferation in the VZ

**Figure 6:**
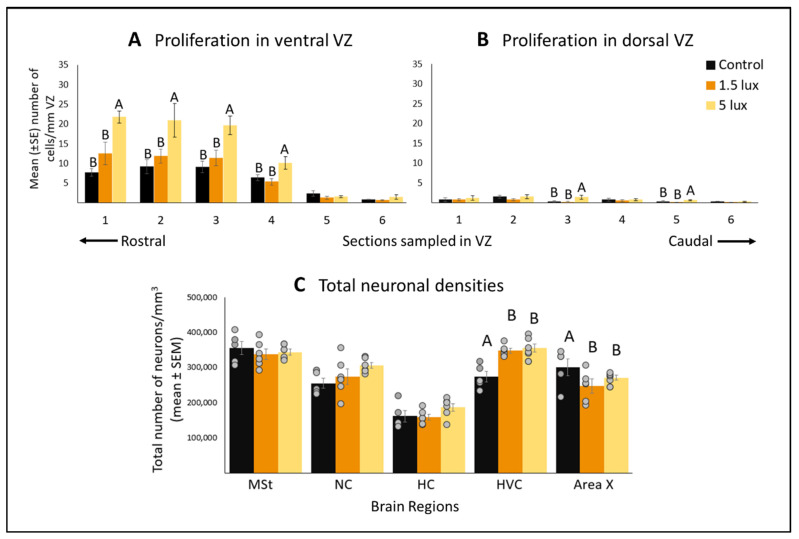
Cell proliferation (number of cells/mm; mean ± SE) in ventral (**A**) and dorsal (**B**) walls of the VZ, in brains of birds exposed to ALAN (1.5 and 5 lux) and controls that were exposed to complete darkness at night. Six sections were sampled along the rostral-caudal axis of the VZ (for details see Figure 4). Total neuronal densities (number of neurons/mm^3^; mean ± SE; (**C**)) in the brain regions MSt (medial striatum), NC (nidopallium caudale), HC (hippocampus), HVC, and Area X, of birds exposed to 1.5- and 5 lux ALAN, and controls that were exposed to complete darkness at night. Grey dots indicate individual data points, different letters indicate significant differences (Tukey post hoc tests, following one-way ANOVAs within each section). *n* = 6 birds per group. When no letters are mentioned, there is no difference among groups.

Exposure to ALAN increased CP in males (F_(3,15)_ = 8.51; *p* = 0.0009; mixed model analyses of the means of the values for all six sections, and dorsal and ventral VZ as the repeated factor; Figure 6A,B). This increase was present in both the ventral (F_(3,15)_ = 6.83, *p* = 0.0007) and the dorsal walls (F_(3,15)_ = 8.99, *p* = 0.0014). This increase was significant only when exposing the birds to 5 lux: in sections 1–4 of the ventral wall (Figure 6A), and in sections 3 and 5 of the dorsal wall (Figure 6B).

### 3.2. ALAN Exposure Differentially Affects Total Neuronal Densities in Some Brain Regions after Short Term Exposure

In the CP experiment, in which birds were exposed to three weeks of ALAN, total neuronal densities increased in the HVC (F_(2,15)_ = 7.72, *p* = 0.0245) and decreased in Area X (F_(2,15)_ = 6.46, *p* = 0.0332) in both the 1.5 lux and the 5 lux groups compared with control, whereas the MSt, NC and the HC were not affected by either of the ALAN intensities (Figure 6C).

### 3.3. ALAN Increases New Neuronal Recruitment in Some Brain Regions and Subregions

**Figure 7:**
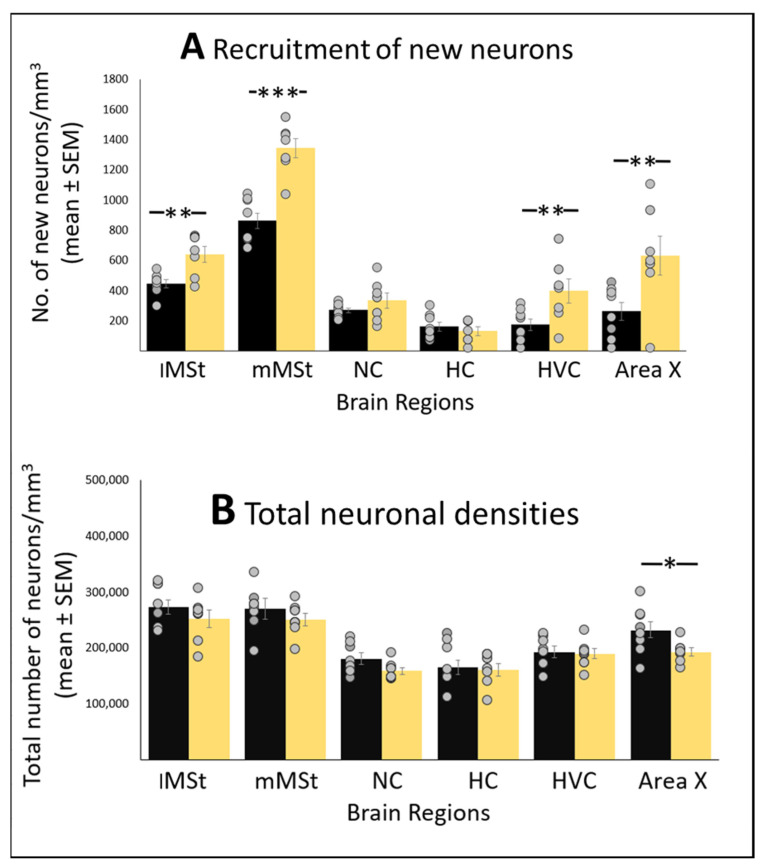
(**A**) Neuronal recruitment (number of new neurons mm^3^; mean ± SE) and (**B**) Total neuronal densities (number of neurons/mm^3^ ± SE) in the lMSt (lateral medial striatum), mMSt (medial medial striatum), NC (nidopallium caudale), HC (hippocampus), HVC and Area X, in brains of birds exposed to 5 lux ALAN, and controls that were kept under complete darkness at night. Grey dots indicate individual data points; *, **, ***—indicate significant differences (*p* < 0.05, *p* < 0.005, and *p* < 0.0005 respectively; Tukey post hoc tests following one-way ANOVAs for each section separately). *n* = 8 birds per group.

Overall, exposure to 5 lux ALAN increased the recruitment of new neurons (F_(1,14)_ = 8.46, *p* = 0.0005; mixed model analyses of the combined data for all brain regions; Figure 7A). The increase was significant compared with control in the lMSt (F_(1,14)_ = 4.83, *p* = 0.0061), mMSt (F_(1,14)_ = 3.80; *p* = 0.0009), HVC (F_(1,14)_ = 4.54, *p* = 0.0042) and Area X (F_(1,14)_ = 4.48, *p* = 0.007), but not in the NC (F_(1,14)_ = 4.07, *p* = 0.1871) and the HC (F_(1,14)_ = 1.07, *p* = 0.1442).

### 3.4. ALAN Decreases Total Neuronal Densities But Only in Area X after Long Term Exposure

Total neuronal density decreased significantly in Area X of birds that were exposed to ALAN for six weeks (NR experiment) compared with control (F_(1,14)_ = 9.72, *p* = 0.0121; Figure 7B). No effect of ALAN on total neuronal densities was observed in any of the other four regions.

### 3.5. Total Neuronal Densities Relative to the Control Decreased in the HVC as a Function of ALAN Exposure Duration

**Figure 8:**
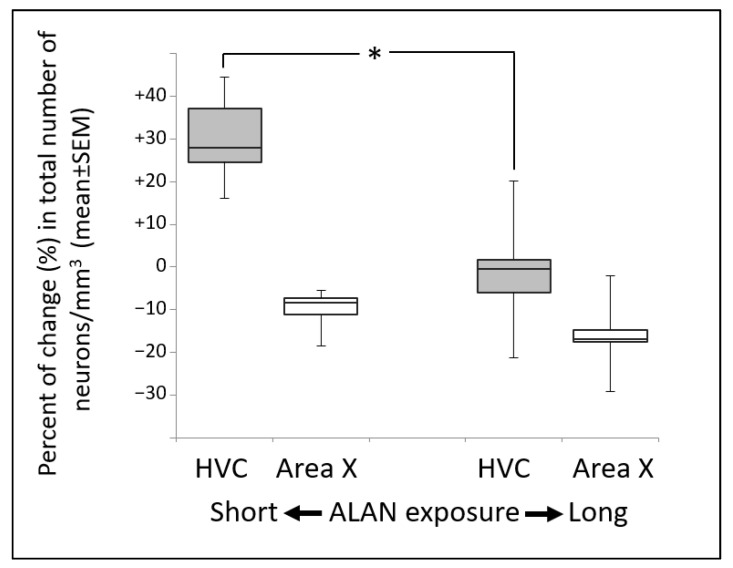
Total neuronal densities (adjusted to controls; number of neurons/mm^3^ mean ± SE) in HVC and Area X, in brains of birds exposed to short (three weeks) and long (six weeks) term 5lux ALAN. *—indicates a significant difference of *p* < 0.0001; Tukey post hoc tests following one-way ANOVAs for each brain region separately. *n* = 8 birds/group.

The effect of ALAN exposure duration on total neuronal densities was compared in the two regions related to vocalization, the HVC and Area X. When adjusted to controls, total neuronal densities in the HVC was lower in birds that were exposed to longer duration of ALAN exposure (six weeks) compared with short duration (three weeks; F_(3,15)_ = 4.55, *p* = 0.0001; Figure 8). We did not detect an effect of ALAN duration on the total neuronal densities in Area X (F_(1.14)_ = 11.55, *p* = 0.0824; Figure 8).

### 3.6. ALAN Reduces Nocturnal Melatonin Levels

**Figure 9:**
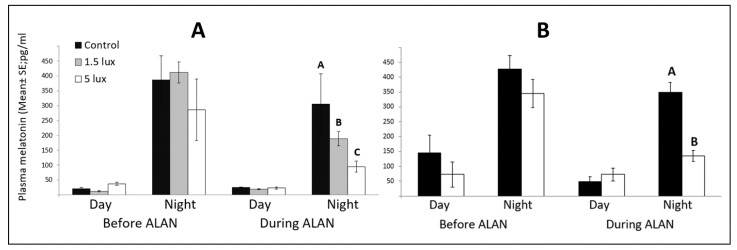
Day and night plasma melatonin levels (pg/mL; Mean ± SE) before and after exposure to ALAN (1.5, 5 lux), compared with controls that were kept under dark nights in (**A**) proliferation (n = six birds per group); and (**B**) neuronal recruitment (n = eight birds per group) experiments. Different letters indicate significant differences (*p* < 0.05, Tukey post hoc tests following one-way ANOVAs between groups). Groups with no letters are not significantly different.

Diurnal and nocturnal MEL plasma concentrations before and during ALAN exposures are presented in Figure 9. Overall, as expected, diurnal MEL levels were lower than the nocturnal ones in both the CP and the NR experiments (F_(2,14)_ = 151.557, *p* = 0.0091, and F_(1,20)_ = 216.557, *p* = 0.0042, respectively). Similarly, in each experiment, before ALAN exposure, when all groups experienced complete dark nights, nocturnal MEL levels were similar across groups. However, in both experiments, when the experimental groups were exposed to ALAN, nocturnal MEL levels were reduced compared to the controls (F_(2,20)_ = 2.618, *p* = 0.0111 in the CP experiment, Figure 9A; and F_(1,14)_ = 3.425, *p* = 0.0223 in the NR experiment; Figure 9B).

### 3.7. ALAN Does Not Affect Body Mass

No differences were found in the body mass between the ALAN and control groups, before and after exposure to ALAN for either three or six weeks. Body weights were 13.4 ± 0.32 g in the control group; 13.5 ± 0.31 g in the 1.5 lux ALAN group; 13.3 ± 0.33 g in the 5 lux ALAN group (n = six birds per group) after three weeks exposure (F_(1,19)_ = 1.59, *p* = 0.8142); 12.4 ± 0.31 g in the control group; and 13.1 ± 0.32 g in the 5 lux ALAN group (n = eight birds per group) after six weeks (F_(1,15)_ = 2.56, *p* = 0.7141).

## 4. Discussion

### 4.1. ALAN Increases Cell Proliferation in the VZ

Overall, in all three groups (control, 1.5 lux, and 5 lux), CP in the ventral VZ wall was higher than in the dorsal wall, similar to what was previously found in canaries (*Serinus canaria*) [49], and in female zebra finches [6].

The ALAN intensities that we used in the present study, 1.5 and 5 lux, were chosen because of their ecological relevance [49]. However, exposure of male zebra finches to 1.5 lux ALAN did not affect CP, unlike the increase that we previously observed in females which were kept under identical conditions [6]. Only exposure to 5 lux ALAN increased CP in specific locations along the rostral-caudal axis of the VZ in males in both walls (although more so in the ventral). This result echoes our findings in females [6]. Our previous suggestion that an increase in cell proliferation might be caused by perturbation of the circadian rhythm under ALAN, and is mediated by MEL [6], might hold here as well. However, this suggestion does not explain why in males, unlike females, no differences were observed under 1.5 lux ALAN. The fact that in males only the higher ALAN intensity affected cell proliferation might indicate that in this respect, males are more resilient to ALAN than females. At this stage, we cannot explain why. However, if intersexual differences occur also in other avian species, it should be taken into account when conducting ecological studies.

In the ventral wall of the VZ, the increase of cell proliferation under exposure to 5 lux ALAN was significant in the four more rostral sections. This seems to be a shift rostrally, compared to the pattern that we previously found in females ([6], upper panel in Figure 4 there). A rostral-caudal pattern of cell proliferation in the VZ was already reported in songbirds (canaries [50]), and was explained by the need to supply new neurons to nearby neurogenic brain regions. Accordingly, the more rostral increased proliferation in the ventral VZ in males might be due to Area X, which exists only in males, and is located in the rostral part of the brain (Figure 5) and corresponds to our Section 1 (Figure 4). However, in the dorsal VZ wall, the increased proliferation under ALAN in males seems to occur more caudally compared to that in females ([6], upper panel in Figure 4), although the same brain regions (such as the HC) are located dorsally to it in both sexes. Therefore, the explanation that increased proliferation answers the need to supply new neurons to nearby regions might not hold.

### 4.2. ALAN Increases Neuronal Recruitment in Some Brain Regions

Overall, exposure of males to 5 lux ALAN caused an increase in new neuronal recruitment in several brain regions. The two nuclei that are part of the song-system and are male-specific, HVC and Area X, seem to be sensitive to ALAN, as evident by the increase in neuronal recruitment in these brain regions (Figure 7A). HVC is involved in vocal learning and song production [51], and Area X is important for song learning and maintenance [52,53]. Therefore, any changes in the homeostasis and functionality of these two regions might affect song production and structure, and that, in turn, might have direct fitness consequences [21]. It could be that the effect of ALAN on neuronal recruitment in these regions is mediated by the observed suppression of nocturnal MEL levels. This is because MEL receptors were found in the song control system in several bird species [54,55,56], and in European starlings (*Sturnus vulgaris*) exogenous MEL attenuated the long-day-induced volumetric increase in HVC [57]. Taken together, these evidences suggest a functional role for MEL in brain plasticity.

MSt (which includes lMSt and mMSt) exhibited increased recruitment in males. The MSt is part of the somatomotor basal ganglia in birds, plays a role in visual perception, and receives inputs from the substantia nigra pars compacta and from regions involved in somatosensory, visual, auditory, and motor function, as reviewed in [7]. Therefore, we previously suggested [7] that the increased recruitment in this region under ALAN might be due to increased nocturnal locomotor activity, caused by sleep disruption, which has been reported in various avian species including zebra finches, e.g., [48]. Support for this suggestion comes also from our yet-unpublished behavioral data, indicating that zebra finches that are exposed to ALAN are more active during the night than control birds that are kept under dark nights.

**Figure 10:**
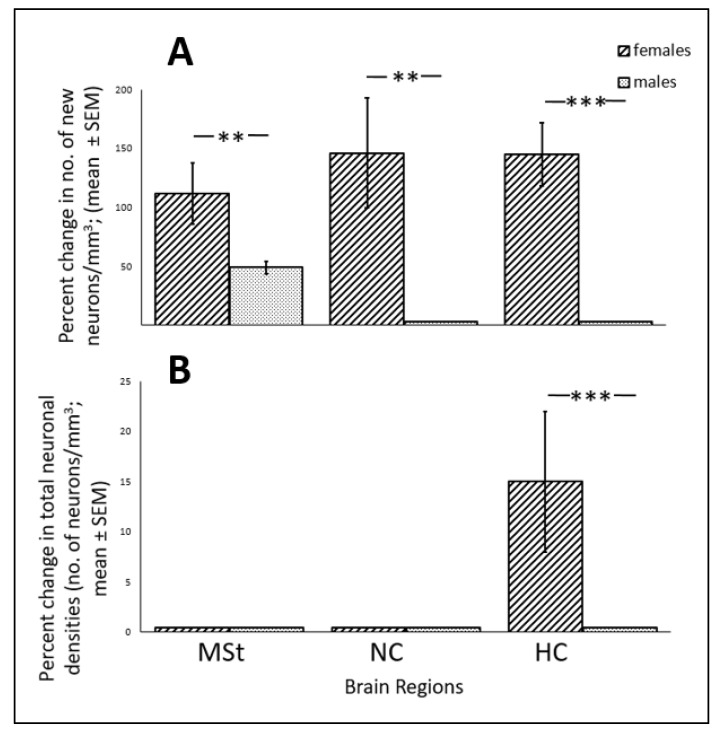
(**A**) Percent change in neuronal recruitment (number of new neurons/mm^3^; mean ± SE) compared to corresponding control, and (**B**) percent change in total neuronal densities (number of neurons/mm^3^ ± SE), in MSt, NC and HC, in brains of female and male zebra finches exposed to 5 lux ALAN for 6 weeks. Female data adapted from Ref. [7]. N = 8 for each sex. **, ***—indicate significant differences (*p* < 0.005, and *p* < 0.0005 respectively; Tukey post hoc tests following one-way ANOVAs for each section separately).

MSt exists in both sexes, and in a previous study [7] we found a similar effect of increased recruitment of new neurons also in females. However, it is interesting to note that the effect was more robust in females than in males, and this difference is demonstrated in Figure 10A: 110% more than the corresponding control (calculated from Figure 1 in [7]), vs. 49% (based on Figure 7A in the present study), accordingly. This difference might indicate that the MST of males is more resilient to ALAN than that in females. Another indication that some brain regions in males might be more resilient to ALAN than those in females, is that neuronal recruitment in the two other tested brain regions (NC and HC) was not affected by ALAN in males, unlike our previous findings in females [7]. This sex difference which is overall significant (F_(1,15)_ = 15.848; *p* < 0.0005), is also demonstrated in Figure 10A.

### 4.3. Differential Effects of the Duration of Exposure to ALAN on Total Neuronal Densities

In birds, new neurons that are recruited in various brain regions replace older ones that die [13,58], and the assumption is that the total number of neurons within a region remains constant. In three of the tested regions (MSt, NC, and HC) we did not observe any change in total neuronal densities compared to controls, both in short (three weeks; Figure 6C) and in long-term (six weeks; Figure 7B) exposure to ALAN, despite the increased NR in MSt, possibly due to higher turnover rate in these regions under ALAN. By contrast, in the two male-specific regions (HVC and Area X) ALAN affected total neuronal densities. Although both HVC and Area X are part of the song system in birds [31,32], the effect of ALAN on their total densities varied. In HVC there was a temporary increase in the short-term exposure (Figure 5), which disappeared in the longer term, as neuronal densities were then comparable to control levels (Figure 7B and Figure 8). This outcome, which occurred despite the increase in neuronal recruitment, indicates a possible faster neuronal turnover. By contrast, Area X was the only region that did not retain its total densities under ALAN, which decreased compared to controls both in the short- (Figure 6C) and the long-term (Figure 7B and Figure 8) exposure. This outcome might be because apoptosis exceeded neuronal recruitment, despite the fact that the latter increased compared to controls. We currently do not have an explanation to these varied effects of ALAN on total densities in the two regions that are part of the song-system, and future studies should record singing behavior under ALAN and its potential effects on mating success. Investigating song production and structure under ALAN conditions might be interesting since, as mentioned above, MEL binding sites were found in song control nuclei of several species, and there is a strong relation between MEL and song [59]. Such information will help to understand the interplay between ALAN, MEL, neuronal plasticity, and song production, and might yield information of ecological relevance regarding conservation of bird species.

An intersexual comparison regarding the effect of ALAN on total neuronal densities raises again the possibility that males are more resilient than females (as indicated in Figure 10B; F_(1,15)_ = 4.848; *p* < 0.02). This is due to the fact that, unlike the situation in males, in which total densities in MSt, NC, and HC remained similar to controls both in the short (Figure 6C) and long-term (Figure 7B and Figure 10B), in females we observed a decrease in the NC and the MSt in the short-term (Figure 4 in [6]) and an increase in HC in the long-term in females (Figure 3 in [7]). These differential sex effects on total densities, despite the similar positive effects on cell proliferation and neuronal recruitment, might indicate different levels of apoptosis between the sexes.

### 4.4. Effects of ALAN on Nocturnal Melatonin Levels

MEL levels in our control groups were comparable to previously published data for this species [60]. In all groups that were exposed to ALAN, nocturnal MEL levels were lower compared to the controls that were exposed to dark nights, and this is in line with the MEL suppression previously observed by others in great tits [61], and in female zebra finches [6,7]. Under control (dark nights) conditions, females and males had similar MEL levels (417 ± 22 pg/mL and 407 ± 19.5 pg/mL, respectively; the females’ concentration is calculated from the means of the control groups before ALAN exposure in [6,7], and the males’ concentration is calculated from the respective means in the present study). This indicates that under normal conditions there are no intersexual differences in MEL levels. However, when exposed to ALAN, there seems to be a difference between the sexes in their response to various intensities. Females showed about a 50% decrease in MEL levels between control and both 1.5 and 5 lux ALAN, with no difference between the two latter ones [6]. However, males exhibited a more gradual decrease, of about 30% between control and 1.5 lux ALAN, and then a 45% decrease between 1.5 and 5 lux ALAN (this study). This difference supports our suggestion that males are more resilient to ALAN, at least in low ALAN intensities.

## 5. Conclusions

Our study indicates that ALAN affects the brains of male zebra finches. This is demonstrated by the increased CP in the VZ and increased NR in several brain regions, compared to controls that were kept under dark nights. In addition, we found, for the first time, that song nuclei were highly affected by ALAN, which caused an increased in NR in these regions. In line with our previous observations in females, we can conclude that ALAN similarly affects both sexes in zebra finches. However, there were several indications that males might be more resilient to ALAN exposure than females, for example in regards to the effect of ALAN on nocturnal MEL levels, which were affected less in males than in females by low intensity. The effects of the duration to ALAN exposure on total neuronal densities in the studied regions varied between the sexes and between the song nuclei in males, suggesting different levels of apoptosis. Taken together, these findings emphasize the importance of studying both sexes when considering environmental effects on brain plasticity and lay the ground for the next challenges in our understanding of the underlying mechanisms of ALAN and MEL effects on neuronal plasticity and, in turn, on behavior. We believe that more work should be done in both sexes in order to complete the picture. For example, our results do not distinguish how ALAN is perceived by the birds, whether it is through the eyes or the pineal gland. This question can be tested by experimentally blocking the pineal gland, or by using monochromatic light that is less likely to reach through the skull. Other interesting future directions include recording apoptosis, exposing birds to various durations of ALAN (including life-long exposure), and investigating various behavioral patterns such as nocturnal activity and song.
